# Ventilator-associated pneumonia prevention in the Intensive care unit using Postpyloric tube feeding in China (VIP study): study protocol for a randomized controlled trial

**DOI:** 10.1186/s13063-022-06407-5

**Published:** 2022-06-09

**Authors:** Linhui Hu, Kaiyi Peng, Xiangwei Huang, Zheng Wang, Quanzhong Wu, Yumei Xiao, Yating Hou, Yuemei He, Xinjuan Zhou, Chunbo Chen

**Affiliations:** 1 Department of Critical Care Medicine, Maoming People’s Hospital, 101 Weimin Road, Maoming, 525000 Guangdong China; 2 Department of Clinical Research Center, Maoming People’s Hospital, 101 Weimin Road, Maoming, 525000 Guangdong China; 3 Department of Surgical Intensive Care Unit, Maoming People’s Hospital, 101 Weimin Road, Maoming, 525000 Guangdong China; 4 Department of Neurocritical Care Unit, Maoming People’s Hospital, 101 Weimin Road, Maoming, 525000 Guangdong China; 5 Department of Oncology, Maoming People’s Hospital, 101 Weimin Road, Maoming, 525000 Guangdong China; 6 Department of Intensive Care Unit of Cardiovascular Surgery, Guangdong Cardiovascular Institute, Guangdong Provincial People’s Hospital, Guangdong Academy of Medical Sciences, 96 Dongchuan Road, Guangzhou, 510080 Guangdong China; 7 Department of Critical Care Medicine, Guangdong Provincial People’s Hospital, Guangdong Academy of Medical Sciences, 106 Zhongshan Er Road, Guangzhou, 510080 Guangdong China; 8 The Second School of Clinical Medicine, Southern Medical University, Guangzhou, 510080 Guangdong China

**Keywords:** Ventilator-associated pneumonia, Prevention, Postpyloric tube feeding, Randomized controlled trial, Efficacy, Safety

## Abstract

**Background:**

Ventilator-associated pneumonia is a challenge in critical care and is associated with high mortality and morbidity. Although some consensuses on preventing ventilator-associated pneumonia are reached, it is still somewhat controversial. Meta-analysis has shown that postpyloric tube feeding may reduce the incidences of ventilator-associated pneumonia, which still desires high-quality evidence. This trial aims to evaluate the efficacy and safety profiles of postpyloric tube feeding versus gastric tube feeding.

**Methods/design:**

In this multicenter, open-label, randomized controlled trial, we will recruit 924 subjects expected to receive mechanical ventilation for no less than 48 h. Subjects on mechanical ventilation will be randomized (1:1) to receive postpyloric or gastric tube feeding and routine preventive measures simultaneously. The primary outcome is the proportion of patients with at least one ventilator-associated pneumonia episode. Adverse events and serious adverse events will be observed closely.

**Discussion:**

The VIP study is a large-sample-sized, multicenter, open-label, randomized, parallel-group, controlled trial of postpyloric tube feeding in China and is well-designed based on previous studies. The results of this trial may help to provide evidence-based recommendations for the prevention of ventilator-associated pneumonia.

**Trial registration:**

Chictr.org.cn ChiCTR2100051593. Registered on 28 September 2021

**Supplementary Information:**

The online version contains supplementary material available at 10.1186/s13063-022-06407-5.

## Background

Ventilator-associated pneumonia (VAP) is defined as an infection of the pulmonary parenchyma in patients exposed to invasive mechanical ventilation for at least 48 h. It is one of the significant nosocomial infections in intensive care units (ICUs), contributing to increased ICU stay, morbidity, and mortality [1, 2]. Zhang et al. [1] recently reported the standardized VAP incidence and mortality of 33.7% and 34.5% in China. Many host- and treatment-related colonization factors, such as the severity of the patient’s underlying disease, prior surgery, exposure to antibiotics, and exposure to invasive respiratory devices and equipment, are essential in the pathogenesis of VAP [3–5]. Intravenous antimicrobial therapy is the cornerstone of VAP treatment, emphasizing prompt empiric treatment and early initiation of pathogen-specific treatment with appropriate duration [6–9]. However, despite the advances in the understanding and management of VAP over the past decade, the disease is associated with a significant economic burden and poor outcomes in ICU patients. In a systematic review, VAP conferred a twofold attributable risk of dying in the ICU, with an assignable cost ranging from USD$10,000 to $13,000 per patient [10]. Therefore, preventing VAP before it occurs is also a patient safety priority.

In addition to avoiding intubation and speeding extubation as the prevention strategies of VAP, a bundle of measures, which include elevation of the head of the bed, daily sedation vacations, assessment of readiness to extubate, deep-vein thrombosis prophylaxis, and daily oral care, are also considered standard of care [11]. However, VAP prevention strategies are variably applied in clinical practice [12–14], underscoring the need for reliable, safe, effective, and available VAP reduction strategies. As we know, enteral nutrition has been considered a modifiable risk factor for VAP development, mainly because of an increased risk of aspiration of gastric contents [15, 16]. The stomach may be one of the potential reservoirs of nosocomial pathogens that contribute to bacterial colonization via aspiration into the lower respiratory tract [3, 17–22]. Emerging as a clinically plausible strategy, postpyloric tube feeding (PTF) may represent a novel approach to preventing VAP through influencing microbiota, enhancing gut barrier function, and reducing gastric reflux and aspiration of gastric content [23–27]. Systematic reviews suggest that PTF reduces VAP by 53–55% compared with gastric tube feeding (GTF) [26, 28]. Nevertheless, most previous randomized trials are small-sample-sized, leading to a less persuasive conclusion. Meta-analyses of small and weak-powered trials often yield implausibly large treatment effects [25, 27]. Hence, the clinical benefits of PTF may be underestimated, and a large, well-powered multicenter trial is needed.

Despite widely accepted recommendations for PTF in nutrition delivery in ICU patients, few studies have assessed the ability of this intervention for VAP prevention. We, therefore, designed this prospective, multicenter, open-label, randomized control trial to validate the efficacy and safety of enteral nutrition via PTF inserted by endoscopy versus GTF in lowering VAP incidence, shortening mechanical ventilation days, ICU or hospital stay, and improving patient outcomes including mortality.

### Objectives

#### Primary objectives

The primary objectives of this trial are to:

1. Determine the efficacy of PTF in lowering VAP incidence in critically ill patients receiving mechanical ventilation for more than 48 h
2. Determine the safety of PTF in lowering VAP incidence in critically ill patients receiving mechanical ventilation for more than 48 h

#### Secondary objectives

The secondary objectives of this trial are to:

1. Determine the efficacy of PTF in improving mortality in critically ill patients receiving mechanical ventilation for more than 48 h
2. Determine the efficacy of PTF in shortening mechanical ventilation days, ICU or hospital stay, and if there is a benefit to decreasing the whole care cost in critically ill patients receiving mechanical ventilation for more than 48 h
3. Determine the efficacy of PTF in improving nutrition deficit and immune ability to infection in critically ill patients receiving mechanical ventilation for more than 48 h

## Methods/design

### Study design

The VIP study is designed as a prospective, multicenter, open-label, randomized, parallel-group, GTF-controlled (PTF vs. GTF 1:1) superiority trial in 4 tertiary care hospitals. The participating sites in this study include medical/surgical, medical, surgical, or emergency ICUs (see Table 1 for details of participating centers). Patient enrollment is expected to last for up to 36 months. The end of the study is defined as the last follow-up of the last enrolled patient. The trial has been registered at chictr.org.cn (ChiCTR2100051593). We used the Standard Protocol Items: Recommendations for International Trials (SPIRIT) reporting guidelines [29], and the SPIRIT checklist is attached in the Supplementary appendixes.

**Table 1:** Research settings and names of each ethics committee

Research setting	Ethics committee name	Hospital rank and location
Maoming People’s Hospital	Medical Ethics Committee of Maoming People’s Hospital	Tertiary, Maoming, Guangdong, China
Guangdong Provincial People’s Hospital	Medical Ethics Committee of Guangdong Provincial People’s Hospital	Tertiary, Guangzhou, Guangdong, China
General Hospital of Southern Theater Command	Medical Ethics Committee of General Hospital of Southern Theater Command	Tertiary, Guangzhou, Guangdong, China
Heyuan People’s Hospital	Medical Ethics Committee of Heyuan People’s Hospital	Tertiary, Heyuan, Guangdong, China

### Patient and public involvement

Unlike patients living with long-term medical conditions such as diabetes and hypertension, most ICU patients unanticipated being admitted into ICU. They are relatively elder and lack clinical research education and experience, making it challenging to determine who will be the potential beneficiaries in attending our trial. However, we have planned to get our participants involved in the communication and education during the research process to increase awareness and knowledge, build confidence and control with self-management, and finally prevent and cure VAP.

### Recruitment

A well-trained study coordinator in each participating center that fulfills the requirement that a gastroscope can be performed at the bedside will be responsible for screening all potentially eligible patients based on the eligibility criteria. After confirming the patient’s eligibility for the trial, the study coordinator will obtain written informed consent from the patient or authorized representatives.

### Inclusion criteria

Patients are eligible for the trial if they are expected to receive mechanical ventilation for no less than 48 h, are over the age of 18 years, and require GTF [30]. Patients will be enrolled within 6 h of their endotracheal intubation.

### Exclusion criteria

Patients will be excluded if they are intubated for more than 6 h, have contradictions for feeding tube placement, or present conditions which will preclude the diagnosis, monitoring, and assessment of VAP development. Detailed exclusion criteria are summarized in Supplementary Appendix 1.

### Drop-out criteria

Patients who meet eligibility criteria and write an informed consent form (see Supplementary Appendix) to participate but fail to accomplish the study process are regarded as drop-out cases if they meet any of the following criteria: (1) the participant or the legal representative requests withdrawal at any time, (2) the investigator considers it inappropriate to continue for safety purpose, and (3) additional reasons for the participant to discontinue from an investigator’s medical perspectives. The drop-out rate must be no more than 10% in this trial.

### Diagnosis of ventilator-associated pneumonia and adjudication process

VAP should rather be suspected in patients with clinical signs of infection, such as at least two of the following criteria: new onset of fever, purulent endotracheal secretions, leukocytosis or leucopenia, increase in minute ventilation, a decline in oxygenation, or increased need for vasopressors to maintain blood pressure [31].

In cases of suspected VAP, the Clinical Pulmonary Infection Score (CPIS) and Sequential Organ Failure Assessment (SOFA) score are assessed. Bedside anteroposterior chest radiography, arterial blood gas analysis, blood cultures, and quantitative sampling of the lower respiratory tract (by either bronchoalveolar lavage or endotracheal aspiration, at the discretion of the attending physician) [32] are performed before any antibiotics are administered.

An adjudication committee composed of one senior radiologist and two senior intensivists unaware of the trial-group assignments reviewed all patients’ medical charts and adjudicated all respiratory tract infections. The intensivists have access to all monitored data, chest radiographs obtained during the ICU stay, and microbiologic documentation. Two of them analyzed data from every patient independently, and in case of disagreement, the third one arbitrated the diagnosis of VAP. Such infections are defined as early if they occurred within 7 days after randomization and as late if they occurred after 7 days, according to an adjudication chart (Supplementary Appendix 2) and the definition of VAP. All secondary infections that happened during the ICU stay are also recorded.

A diagnosis process to confirm reported clinical VAP is defined in a standardized approach with the use of criteria from the 2020 Food and Drug Administration guidance for diagnosis and confirmation of VAP, which relies on clinical, laboratory, and radiologic criteria (patients have to meet all three types of standards). The flow chart for diagnosis and confirmation of VAP is summarized in Supplementary Appendix 3.

### Endotracheal tube, feeding tube, and enteral nutrients

According to Good Manufacturing Practice guidelines, all feeding tubes are prepared by Henan Anesthesia Medical Technology Co, Ltd. All endotracheal lines and enteral nutrients are provided by participating centers according to their routine practices. All studying materials aforementioned are shipped to each participating center by express service for a 14-day supply and stored in a cool, well-ventilated place to avoid direct sunlight.

### Assignment of interventions

#### Sequence generation for allocation

Following informed consent and confirmation of inclusion and exclusion criteria, participants on mechanical ventilation will be randomly assigned in a 1:1 ratio to receiving either PTF or GTF. Allocation sequences are generated by computer-generated random numbers using the R package *blockrand* [33]. Stratified block randomization is employed to avoid inter-group differences (study or control group) due to differences in the source of patients in different hospitals. Each participating center is served as a stratification factor, and then the subjects in each ICU are randomized into other blocks. The subjects will be consecutively assigned when entering the trial. To reduce the predictability of a random sequence, blocking details are provided in a separate document that is unavailable to those who enroll participants or assign interventions.

#### Allocation concealment mechanism

A central randomization system is set up to conceal the allocation to investigators. To prevent early knowledge of treatment assignment and disruption of the assignment sequence, investigators must complete the clinical trial entry form attached to the case report form (CRF) and obtain the informed consent form before disclosing the unique sequence number and assignment group. Randomization methods and block sizes are blinded until all data analyses are completed.

#### Allocation implementation

Each center’s investigator and authorized clinicians will enroll subjects according to the protocol’s eligibility criteria and allocate the newly registered patient into the corresponding group according to the unique number for specific assignments acquired from a randomization squad. The squad fetches the assignment number through a prepared online central randomization system, which will be sent to the investigator via instant messaging software like WeChat once the informed consent form is signed.

### Interventions

Patients who meet the enrollment criteria will be randomized 1:1 to either the PTF or the GTF group, receiving postpyloric or gastric tube feeding, respectively, with other treatments exactly the same.

#### Endoscopic feeding tube placement

A nasogastric feeding tube is inserted by nursing staff for the control group. Currently, in our clinical practice, the postpyloric feeding tube can be established by self-propelled dynamics from the gut with the assistance of prokinetic agents [34–36] and using a rescued bedside tube placement method by ICU physicians when necessary [37]. However, despite the short learning curve [38] and easy availability of the methods mentioned above, especially in the advantage of some decision support tools [39, 40], we otherwise decided to employ the endoscopic approach to establish the postpyloric feeding tube promptly and reliably for the study group. Hence, experienced endoscopists must be equipped when participating centers are recruited. In most ICUs currently in China, no special endoscopists are regularly prepared. In this case, a training course on endoscopic postpyloric tube placement is established to equip the physicians to become experienced endoscopists in the ICU.

Detailed training protocol is attached as Supplementary Appendix 4 in this manuscript. The essential procedures of endoscopic tube placement are as follows:

Patients are provided with oxygen inhalation, and ECG and blood oxygen saturation are monitored. After the patient is narcotized, they take the left lying position. During the operation, no secretion could be left in the mouth. A lubricated nasointestinal tube, approximately 25cm deep, is inserted through the nostrils and sent to the gastric lumen by gastroscopy. The esophagus, stomach, and duodenum are observed to confirm the absence of lesions or obstruction. The head end of the nasointestinal tube is clamped with foreign body forceps, and it is slowly pushed into the descending part of the duodenum to fix the nasointestinal tube. The gastroscope is returned to the gastric cavity, and the foreign body forceps are released into the gastric cavity. After about three times, they are transported to about 20–40 cm below the Treitz ligament. After the nasointestinal tube is fixed, the gastroscope and the nasointestinal tube guidewire are withdrawn.

#### Nutrition support protocol

In both groups, energy goals are set at 25 kcal per kg of ideal body weight per day, and the protein target is 1.2–2.0 g per kg of ideal body weight per day. Glucose control targets are set following international guidelines [41–43]. Incidences in which the patient develops an intolerance to EN (diagnosed when vomiting, diarrhea, abdominal pain, or abdominal distension occurred) are recorded. In these cases, the rate of feeding and EN are gradually reduced as tolerated. If the nutrition goal is not reached within 7 days, parenteral nutrition is provided.

#### Concomitant interventions

Routine use of a bundle of measures for the prevention of VAP (elevation of the head of the bed, daily sedation vacations and assessment of readiness to extubate, and deep-vein thrombosis prophylaxis) and daily oral care are highly recommended, and specific attention is given to standardize patient care [11].

Participants may receive management preventing from VAP following the 2016 Clinical Practice Guidelines for the Management of Adults with Hospital-acquired and Ventilator-associated Pneumonia by the Infectious Diseases Society of America and the American Thoracic Society, mainly including (1) manage patients without sedation whenever possible; (2) interrupt sedation daily; (3) assess readiness to extubate daily; (4) perform spontaneous breathing trials with sedatives turned off; (5) facilitate early mobility; (6) utilize endotracheal tubes with subglottic secretion drainage ports for patients expected to require more than 48 or 72 h of mechanical ventilation; (7) change the ventilator circuit only if visibly soiled or malfunctioning; and (8) elevate the head of the bed to 30–45. If any, the use of all the above maneuvers should be documented in the electronic case report form (eCRF).

### Outcome measurements

Participants will be evaluated clinically and through laboratory testing according to Table 2. The following data will be recorded: demographics, VAP diagnosis, concurrent medical conditions and comorbidities, inclusion and exclusion criteria, the severity of illness and organ dysfunction scores, vital signs and laboratory results, potential confounding co-interventions (life-sustaining therapies and use of sedatives or vasopressors), and outcomes (vital status at ICU and hospital discharge, day 28 and day 90, ICU and hospital length of stay).

**Table 2:** Trial schedule of the VIP study

Timepoint	Baseline	^**a**^Interventions									Termination
	^b^D0	D1	D2, ^c^N1	D..., N...	D4	D5	D6	D7	D8, N7	D…	D28
Eligibility screening	**×**										
Informed consent obtaining	**×**										
Randomization	**×**										
Medical history collection	**×**										
Population data collection	**×**										
Baseline data collection	**×**										
Evaluation of major adverse tube-associated events (MATE)		**×**	**×**								
Evaluation of ^d^EN related adverse events			**×**	**×**	**×**	**×**	**×**	**×**	**×**	**×**	**×**
Evaluation of serious adverse events		**×**	**×**	**×**	**×**	**×**	**×**	**×**	**×**	**×**	**×**
VAP episode		**×**	**×**	**×**	**×**	**×**	**×**	**×**	**×**	**×**	**×**
Microorganisms causing VAP		**×**	**×**	**×**	**×**	**×**	**×**	**×**	**×**	**×**	**×**
Duration of mechanical ventilation		**×**	**×**	**×**	**×**	**×**	**×**	**×**	**×**	**×**	**×**
SOFA score		**×**	**×**	**×**	**×**	**×**	**×**	**×**	**×**	**×**	**×**
CPIS score		**×**	**×**	**×**	**×**	**×**	**×**	**×**	**×**	**×**	**×**
Serum albumin and C-reactive protein		**×**	**×**						**×**		
ICU-acquired infections (bloodstream, urinary tract, catheter-related, and other infections)		**×**	**×**	**×**	**×**	**×**	**×**	**×**	**×**	**×**	**×**
Day calorie target		**×**	**×**	**×**	**×**	**×**	**×**	**×**	**×**	**×**	
Day actual calorie		**×**	**×**	**×**	**×**	**×**	**×**	**×**	**×**	**×**	
Day calorie deficit		**×**	**×**	**×**	**×**	**×**	**×**	**×**			
Ventilator free		**×**	**×**	**×**	**×**	**×**	**×**	**×**	**×**	**×**	**×**
Transfer out of ICU		**×**	**×**	**×**	**×**	**×**	**×**	**×**	**×**	**×**	**×**
ICU survival		**×**	**×**	**×**	**×**	**×**	**×**	**×**	**×**	**×**	**×**
Discharge		**×**	**×**	**×**	**×**	**×**	**×**	**×**	**×**	**×**	**×**
Hospital survival		**×**	**×**	**×**	**×**	**×**	**×**	**×**	**×**	**×**	**×**
28-day survival											**×**

Eligibility screening includes diagnosis, inclusion/exclusion criteria, urine pregnancy test for female, demographics, medical history, and physical examination

*CPIS* Clinical Pulmonary Infection Score, *EN* Eenteral nutrition, *MATE* Major adverse tube-associated event, *SOFA* Sequential Organ Failure Assessment score

^a^Intervention means postpyloric tube feeding

^b^D means day from enrollment

^c^N means day from enteral nutrion

^d^EN-related adverse events include abdominal distension, gastric retention, vomiting, diarrhea, and aspiration of gastric contents

#### Primary efficacy endpoint

The primary efficacy endpoint of this study is the proportion of patients with at least 1 VAP episode.

#### Secondary efficacy endpoints

The secondary efficacy endpoints of this trial include the following:

1. Incidence analysis: including the cumulative VAP incidence and the total number of VAP episodes and numbers and percentages of microorganisms causing VAP
2. Mortality analysis: including ICU, hospital, and day-28 mortality rates
3. Time analysis: including time to VAP onset from mechanical ventilation, delayed time to first VAP occurrence, mechanical ventilation duration, the number of ventilator-free days until day 28, and ICU and hospital lengths of stay
4. Enteral feeding and nutritional status: including the proportions of patients with at least one vomiting episode, prokinetic treatment, and diarrhea; the proportion of patients given 100% of the calorie target; cumulative calorie deficit from day 0 to day 7; and variations in serum albumin during the first week of enteral nutrition
5. Organ functioning: including score variations in Sequential Organ Failure Assessment (SOFA)
6. Inflammatory level: including variations in serum C-reactive protein (CRP) levels during the first week of enteral nutrition
7. ICU-acquired infection: proportions of patients with ICU-acquired infections (bloodstream, urinary tract, catheter-related, and other infections); score variations in CPIS

We plan to collect data on organ dysfunction at baseline and at various time points during the study. All the six domains of the SOFA score, including respiratory, coagulation, hepatic, cardiovascular, renal, and central nervous system, will be documented. Vasopressors, sedatives, and renal replacement therapy will also be reported to assess organ dysfunction. VAP episodes are monitored during the whole study. New-onset VAP is diagnosed if new microbial culture results from distal respiratory specimens are different from those prior, and colonization is excluded.

#### Safety endpoints

The safety outcomes are major adverse tube-associated events (MATEs), including vital sign alert events (defined as HR, RR, or MAP fluctuating beyond the range of ± 15%, or pulse oxygen saturation declining to < 90%), the requirement for sedatives or analgesics during tube placement, vomiting, rhinorrhagia, misplacing into the thoracic cavity, gastrointestinal bleeding or perforation, and so forth.

### Adverse events and serious adverse events

All treatment-related adverse events (AEs) and serious adverse events (SAEs) should be recorded on eCRF. SAEs will be reported to the Institutional Ethics Committee within 24 h of study staff becoming aware of the events. The participants are provided with commercial clinical research insurance by the manufacturer of the study product. Determination criteria for AE and SAE are detailed in Supplementary Appendix 5.

The treating physician will be responsible for determining the causal relationship of the SAE as either definitely, possibly, possibly not, or definitely not study treatment-related, as well as unclassified.

### Data management

Trained staff will perform data management at each center using the Electronic Data Capture (EDC) system (https://www.mmphcrc.com/pdf/medicalHistory/vapcrf.html). The EDC system’s reliability, access control, and traceability will guarantee the quality of trial data management. Data collection will be restricted to those variables necessary to define baseline patient characteristics (demographics, VAP diagnosis, concurrent medical conditions and comorbidities, inclusion and exclusion criteria, severity of illness and organ dysfunction scores, vital signs, and laboratory results), the delivery of the nutrients, potential confounding co-interventions (life-sustaining therapies, and use of vasopressors or sedatives), and outcomes (vital signs at ICU and hospital discharge, day 28, length of stay in ICU and hospital). Randomized participants will be followed until either death or 28 days after randomization, whichever comes first. Study staff will attend follow-up by either direct contact with the patient or the next of kin. Participants who withdraw from the study will be followed up according to the follow-up schedule and analyzed on the ITT principle.

### Data monitoring committee

A Data Monitoring Committee (DMC) will be responsible for data monitoring and blinded analysis. Members of the committee are experts in medicine, biometric statistics, and medical ethics who are independent and have no competing interests in the trial. No interim analysis is planned for this trial. The DMC will audit the trial regularly and advise the Research Ethics Committee in each center, who will also audit and determine to continue, modify, or discontinue the trial.

### Ethics and dissemination

#### Protocol amendments

Protocol amendments will be documented with a brief description of the change and reference (date and number) when changes in the existing protocol significantly affect the safety of subjects, the scope of the investigation, or the scientific quality of the study. The coordinating investigator is obligated to notify this protocol amendments to all the investigators and the reviewing IRB and other relevant parties as appropriate.

#### Consent or assent

The clinical investigator ensures that informed consent is obtained from each research subject before that subject participates in the research study. When the research subject is disabled to consent for critical illness, informed consent is obtained from the authorized surrogates, for which the prior approval from the IRB is a prerequisite. We will only obtain consent to use data and samples for the research question described in this protocol. Thus, we do not intend to use participant data or biological samples in ancillary studies.

#### Access to data

Data from the VIP study will be made available in the future for collaborative research questions. Such requests must be authorized by the principal investigators and the appropriate Human Research Ethics Committees and Human Research Governance Safety Entities.

#### Ancillary and post-trial care

All patients would be treated, monitored, and routinely assessed regarding the VAP development and recovery process in each participating study center in China.

#### Dissemination policy

The findings from the data analysis will be disseminated in various ways, including abstracts, posters and presentations at conferences, and published manuscripts in peer-reviewed journals. These will also be reported to national, provincial, and local governments to inform policy and reports to funding bodies, institutes, and hospitals that participated in and supported the cohort study. Members of the study team will have publishing and authorship rights following the International Committee of Medical Journal Editors requirements for authorship and as described in research agreements.

### Statistical analysis

#### Sample size estimation

Zhang et al. reported the incidence of 33.7% in a meta-analysis and systematic review of 334 publications concerning ICU-acquired pneumonia and VAP in China [1]. According to a meta-analysis by Ouyang et al. [28], a treatment effect with a relative risk reduction (RRR) of 51.4% is observed in ICU patients receiving postpyloric tube feeding (PTF) compared with receiving gastric tube feeding (GTF). A 10% inferiority margin is predetermined following previous guidelines and reviews. We calculate that enrollment of 924 participants will have a power of 80% to detect an absolute reduction of 17.3% (relative risk reduction of 51.4%) in the study group compared to 33.7% of the control group, allowing a loss of follow-up or withdrawal of 10%. Considering the prevalence of VAP in our patients and assuming the rate of patient recruitment of about ten per month per center (with three participating centers), this trial will be finished within 3 years.

#### Analyses set

The study protocol mention that analysis will be done considering the “intention to treat” principle. However, some randomized patients did not allow feeding tube insertion (gastric or postpyloric) during study conduction due to illness severity, or the attending physician decided not to insert the tubes. Those cases are not excluded from the analyses. Patients who complete the randomization, regardless of whether they completed the trial or received the treatment in the designated group, are retained in the original group for analysis. The randomization information is maintained to the maximum extent.

#### Intention-to-treat set (ITTS)

ITTS refers to the ideal population of subjects meeting the ITT principles. To best retain the randomized information, ensure that the differences in trial results are attributed to the differences in treatment, and make the effect of treatment (postpyloric feeding) best assessed, the principle of ITT analysis is adopted. ITT set (ITTS) included all randomized patients, regardless of whether they have received nasogastric tube placement, completed the trial, or received the treatment of this group, all of whom remain in the original group for analysis.

#### Per protocol set (PPS)

PPS refers to all participants who fulfill the eligibility criteria and achieve gastric or postpyloric tube placement per randomization. They have good compliance with the trial protocol, such as being ready to receive treatment and measurement for the primary efficacy endpoints, with the required contents in the CRF filled. PPS is used to analyze the primary efficacy endpoints.

#### Security set (SS)

The SS included all actual cases that have received at least one feeding tube placement after randomization, with the safety endpoints recorded. The incidence of adverse events is denominated by the number of patients in the safety set.

#### Data analysis

All analyses will be performed according to the ITT principle. A *P* value < 0.05 is considered statistically significant. All tests are two-sided with no adjustment for multiple comparisons. Continuous variables are reported as means and standard deviations or medians and interquartile ranges. Categorical variables are reported as proportions. Pearson’s chi-squared test and adjusted multivariable analysis will be applied for the primary outcome. We plan to perform subgroup analyses for the primary outcome for predefined variables: the proportion of patients with at least 1 VAP episode. All the other data, including age, gender, body mass index (weight, height), SOFA score, primary admission diagnosis, coexisting illness, and type of admission, will be presented as descriptive results. Pearson’s chi-squared test will compare incidence or mortality outcomes between groups. The *t*-test will compare total VAP episodes, SOFA scores, time to VAP onset from mechanical ventilation, mechanical ventilation duration, ICU and hospital lengths of stay, inflammatory biomarkers, and function indicators between groups. A paired *t*-test or repeated measures ANOVA will be used to compare baseline and changes during the intervention.

#### Interim analysis

No interim analysis is planned for this trial.

## Discussion

VAP is one of the most frequent ICU-acquired infections associated with prolonged mechanical ventilation and ICU stay. The reported incidences vary widely from 5 to 40%, depending on the setting and diagnostic criteria. The estimated attributable mortality of VAP is around 10%, with higher mortality rates in surgical ICU patients and patients with mid-range severity scores at admission [31]. All the data above highlight reliable measures to prevent or limit VAP from occurring in the ICU.

The practices most consistently associated with lower mortality rates focus on limiting exposure to invasive mechanical ventilation by avoiding intubation and speeding extubation [44]. However, for those patients whose intubation is inevitable to save lives, many of our presumptions about how best to prevent VAP have recently been challenged. Oral care with chlorhexidine and stress ulcer prophylaxis may be harmful. New data affirm the long-held fear that selective oral and digestive decontamination may not be effective in ICUs with high baseline rates of antibiotic resistance. Subglottic secretion drainage may not shorten the duration of mechanical ventilation or ICU length-of-stay as is once thought [45–49]. Furthermore, two recent RCTs showed no significant difference in VAP development among ventilated ICU patients receiving probiotic or monoclonal antibody administration than placebo [50, 51].

Otherwise, PTF has been evaluated as a potentially promising management in preventing VAP due to not only reducing gastrointestinal and respiratory complications like vomiting and gastric distention in critically ill patients and ensuring that the nutritional goals are better achieved [52–54]. However, current evidence demonstrating the efficacy of PTF in the prevention of VAP, including RCTs, is mainly negative. Two studies [23, 27] showed no significant reduction in VAP incidence comparing PTF with GTF. There exist many critical limitations in those studies, including small-sample-sized and conducting in a single center. Statistically, the results from these studies are underpowered, and the conclusion is far less than robust. As a result, the effect of PTF on the incidence of VAP warrants validation by large, well-conducted RCTs in different settings [55].

To address this call, the VIP study is a large-sample-sized, rigorous multicenter randomized trial that aims to determine whether PTF is effective and safe in preventing VAP. In ethical consideration to maximize patients’ benefit, bundling practices, including elevation of the head of the bed, daily sedation vacations, assessment of readiness to extubate, deep-vein thrombosis prophylaxis, and daily oral care, are also implemented by trained investigators according to the study protocol [56–59]. This strategy might result in a relatively lower occurrence of VAP in the control group, which in turn required larger sample size to detect the potential difference in VAP incidence between the intervention and control groups. Based on an elaborate sample size calculation, we aimed to enroll nearly 1,000 patients to powerfully detect the possible underlying profit of PTF over GTF in improving a range of clinical outcomes for ICU patients.

VIP study has several strengths. First, the RCT design can limit the risk of biases related to the presence of confounding factors in evaluating the preventive effects on VAP and mortality. Second, the VIP study includes representation of persons aged from 18 years to 80 years in different hospitals located all over China to enhance the generalizability of the findings. Third, we are also documenting baseline organ malfunctions with coexisting diseases and SOFA scores to understand further the relationship between body frailty and critical care-associated infections. Furthermore, to shorten the time from endotracheal intubation to PTF placement and limit the effects of management heterogeneity among centers on outcome variables, all tubes are placed using endoscopic methods by well-trained intensivists in the present trial.

In conclusion, the VIP study is a large-sample-sized, multicenter, open-label, randomized, parallel-group, controlled trial of PTF in China and is well-designed based on previous studies. The results of this trial may help provide evidence-based recommendations for the prevention of VAP.

## Trial status

This article is based on the study protocol version 2.8 of 30 August 2021. The VIP study started on 1 December 2021. Participants are currently being recruited and enrolled. Recruitment will probably continue until July 2025. Contact: Chunbo Chen, email: gghicu@163.com.

## Supplementary Information

**Additional file 1.** Exclusion criteria for patient screening.

**Additional file 2.** Adjudication chart for ventilator-associated pneumonia.

**Additional file 3.** Criteria for diagnosis and confirmation of VAP.

**Additional file 4.** Training protocol for bedside endoscopic tube placement.

**Additional file 5.** Determination criteria for AE and SAE in VIP study.

**Additional file 6.** SPIRIT checklist.

**Additional file 7.** Informed Consent Form for Participants’ enrolment—English version.

## Data Availability

All the compiled CRFs will be archived. After this study is complete, the final trial dataset and statistical codes will be available from the corresponding authors upon reasonable request, except for participants’ personal information.

## Abbreviations

AE: Adverse event
CPIS: Clinical Pulmonary Infection Score
CRF: Case record form
DMC: Data Monitoring Committee
eCRF: Electronic case report form
EDC: Electronic Data Capture
EN: Enteral nutrition
GTF: Gastric tube feeding
ICU: Intensive care unit
ITT: Intention-to-treat
PPS: Per protocol set
PTF: Postpyloric tube feeding
RCT: Randomized controlled trial
SAE: Severe adverse event
SOFA: Sequential Organ Failure Assessment
SS: Security set
VAP: Ventilator-associated pneumonia
