# Supplementary material for: Ventilator-associated pneumonia prevention in the Intensive care unit using Postpyloric tube feeding in China (VIP study): study protocol for a randomized controlled trial
Source: Trials. 2022 Jun 9;23:478. doi: 10.1186/s13063-022-06407-5 (PMC9178536; doi:10.1186/s13063-022-06407-5)
Supplement: Supplementary file 1 — Additional file 1. Exclusion criteria for patient screening. [file 13063_2022_6407_MOESM1_ESM.docx]

**Exclusion criteria for patient screening**

Patients will be excluded if they meet any of the following criteria:

(1) ongoing pneumonia or gross aspiration identified during direct laryngoscopy for tracheal intubation and confirmed by the presence of lung infiltrates on chest radiographs at admission;

(2) previous lung disease precluding accurate interpretation of chest radiographs;

(3) pregnancy;

(4) immunocompromise (i.e., hematologic neoplasia, solid organ transplant or congenital or acquired diseases that cause significant immunodeficiency including common variable immunodeficiency and human immunodeficiency virus infection and acquired immune deficiency syndrome, and neutropenia < 0.5×10^9^/L);

(5) active gastrointestinal bleeding or acute phase of acute perforation of the digestive tract; (6) severe esophageal varices;

(7) caustic esophageal or gastric injury;

(8) cerebrospinal fluid rhinorrhea or transnasal surgery;

(9) laryngopharyngeal or esophageal stenosis or obstruction that precludes gastroscope or nasogastric tube from being inserted;

(10) allergic to nasal feeding tube made from polyvinyl chloride materials;

(11) history of any major abdominal surgery (i.e., esophageal, gastric, duodenal or pancreatic surgery) or any abdominal surgery within the past month;

(12) severe cardiac or pulmonary deficiency (i.e., severe hypertension, severe arrhythmia, active myocardial infarction, severe heart failure, severe asthma, and severe respiratory failure that cause the person dyspnea when lying flat;

(13) enteral nutrition via a jejunostomy or gastrostomy;

(14) predictable decision of early care limitation (within 7 days);

(15) terminal illness;

(16) participation in another trial within the previous 30 days.
