# Supplementary figures and images for: Ventilator-associated pneumonia prevention in the Intensive care unit using Postpyloric tube feeding in China (VIP study): study protocol for a randomized controlled trial
Source: Trials. 2022 Jun 9;23:478. doi: 10.1186/s13063-022-06407-5 (PMC9178536; doi:10.1186/s13063-022-06407-5)

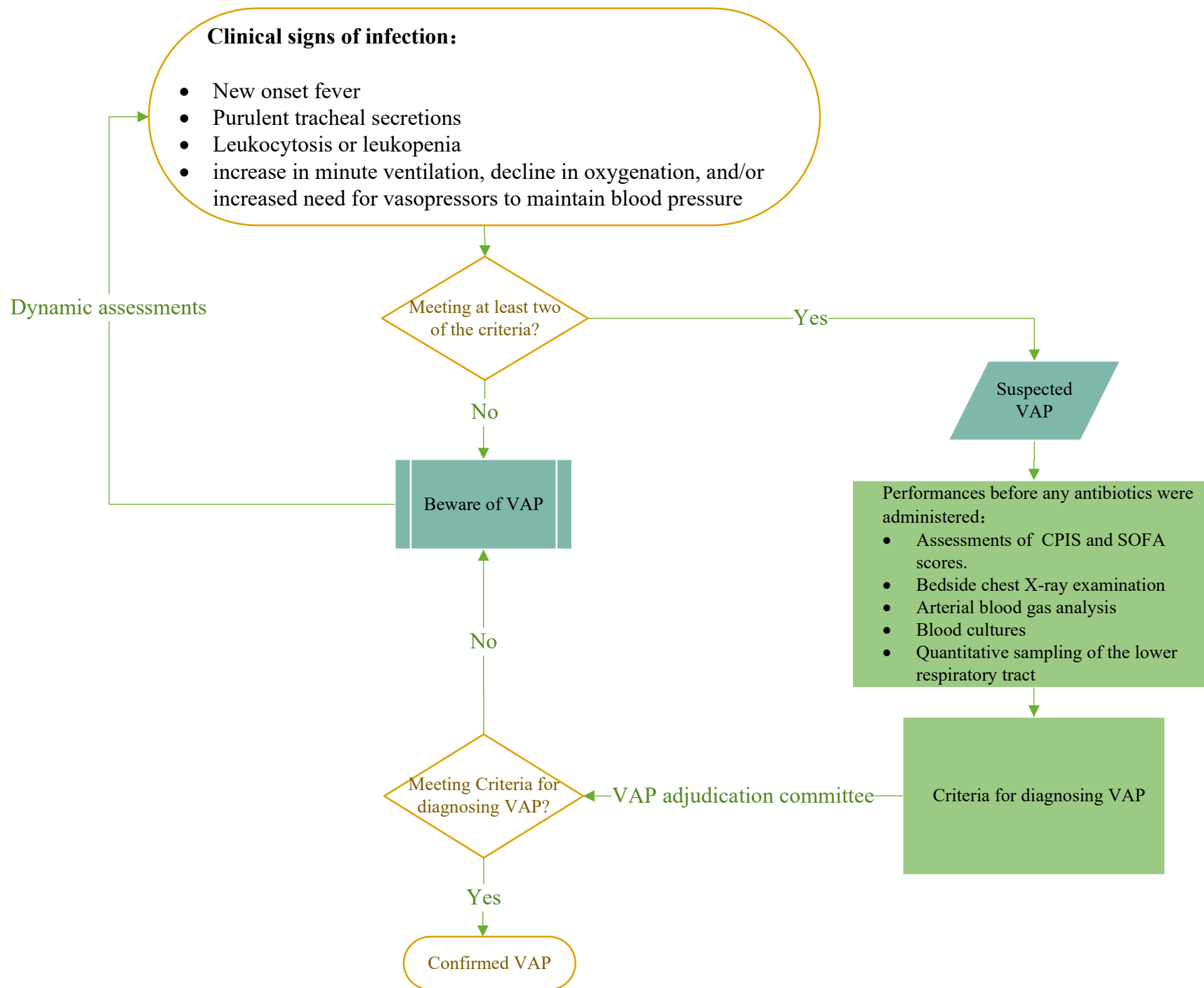

Supplement: Supplementary file 2 — Additional file 2. Adjudication chart for ventilator-associated pneumonia. [file 13063_2022_6407_MOESM2_ESM.pdf]

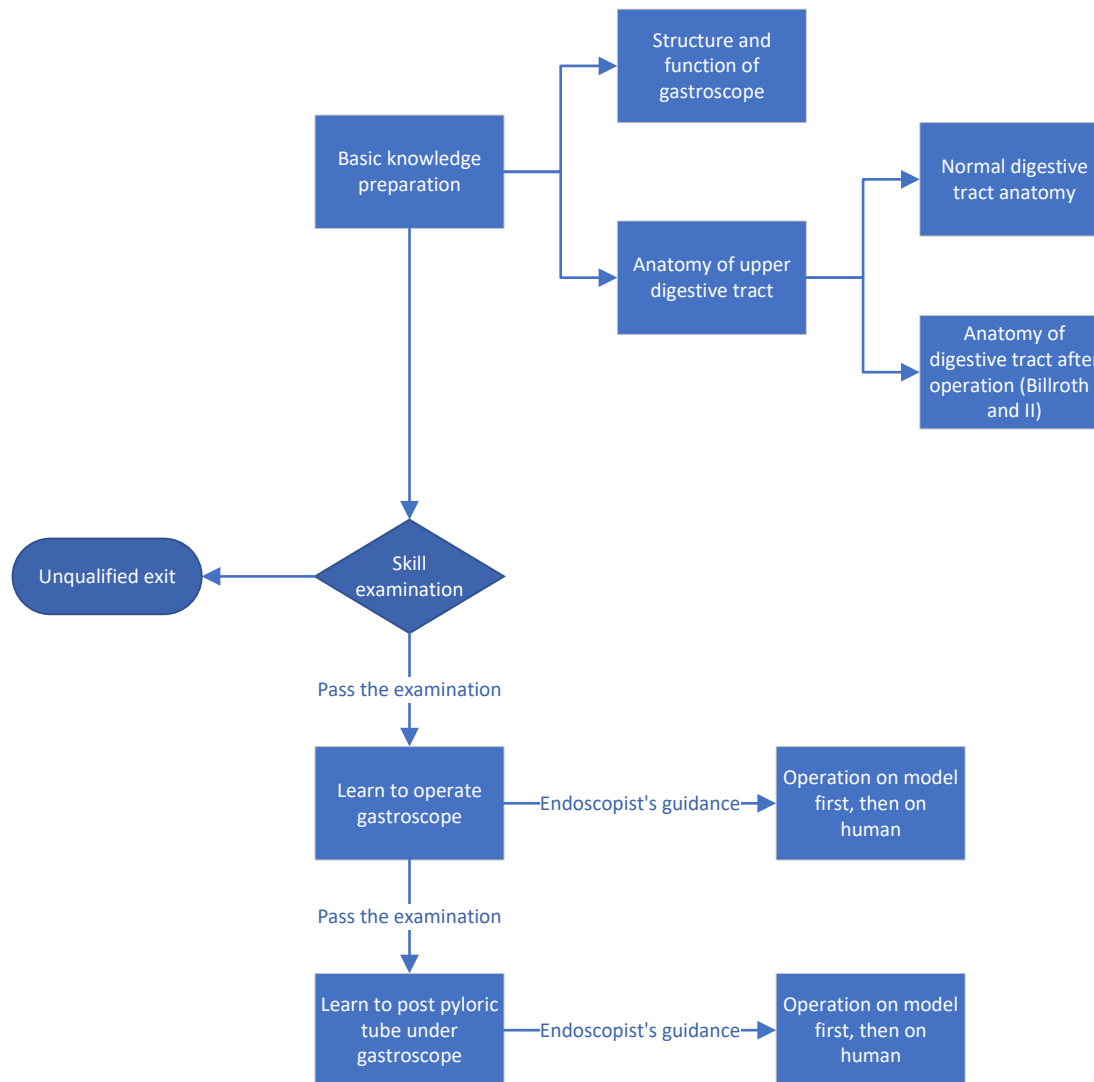

Supplement: Supplementary file 4 — Additional file 4. Training protocol for bedside endoscopic tube placement. [file 13063_2022_6407_MOESM4_ESM.pdf]
