# Supplementary material for: Ventilator-associated pneumonia prevention in the Intensive care unit using Postpyloric tube feeding in China (VIP study): study protocol for a randomized controlled trial
Source: Trials. 2022 Jun 9;23:478. doi: 10.1186/s13063-022-06407-5 (PMC9178536; doi:10.1186/s13063-022-06407-5)
Supplement: Supplementary file 3 — Additional file 3. Criteria for diagnosis and confirmation of VAP. [file 13063_2022_6407_MOESM3_ESM.docx]

**Criteria for diagnosis and confirmation of VAP**

Patients should have at least one of the following clinical features:

• New onset or acute worsening pulmonary symptoms or signs, such as cough, dyspnea, tachypnea (e.g., a respiratory rate greater than 25 breaths per minute), expectorated sputum production, or requirement for mechanical ventilation

• Hypoxemia

• Need for acute changes in the ventilator support system to enhance oxygenation, as determined by worsening oxygenation or needed changes in the amount of positive end-expiratory pressure

• New onset of suctioned respiratory secretions

**In addition**, patients should have at least one of the following signs/laboratory abnormalities:

• Documented fever (e.g., body temperature greater than or equal to 38℃)

• Hypothermia (e.g., core body temperature less than or equal to 35°C)

• Total peripheral white blood cell counts greater than or equal to 10,000 cells per cubic millimeter (mm^3^)

• Leukopenia with total white blood cell count fewer than or equal to 4,500 cells per mm^3^

• Greater than 15 percent immature neutrophils (e.g., bands) noted on peripheral blood smear

**Plus**

• A chest radiograph showing the presence of a new or progressive infiltrate suggestive of bacterial pneumonia
