# Supplementary material for: Ventilator-associated pneumonia prevention in the Intensive care unit using Postpyloric tube feeding in China (VIP study): study protocol for a randomized controlled trial
Source: Trials. 2022 Jun 9;23:478. doi: 10.1186/s13063-022-06407-5 (PMC9178536; doi:10.1186/s13063-022-06407-5)
Supplement: Supplementary file 5 — Additional file 5. Determination criteria for AE and SAE in VIP study. [file 13063_2022_6407_MOESM5_ESM.docx]

**Determination criteria for AE and SAE in VIP study**

The safety profile of endoscopic postpyloric tube placement is an important endpoint of the current study, partially because of the relatively high skill requirements. According to the clinical practice, adverse events (AEs) are uncommon (6%), including vomiting, rhinorrhagia, misplacing into the thoracic cavity, gastrointestinal bleeding or perforation. Furthermore, enteral nutrition-related AEs are also a safety endpoint, including abdominal distension, gastric retention, vomiting, diarrhea, aspiration of gastric contents.

It is recognized that the patient in ICU will experience a number of aberrations in laboratory values, signs, and symptoms due to the severity of the underlying disease and the impact of standard critical medicine therapies. These will not necessarily constitute an AE unless they are considered to be of related to study treatment or a concern in the investigator’s clinical judgement. In this study, reporting of AEs will be restricted to events that are considered to be related to study treatment (possibly, probably or definitely). Serious adverse events (SAEs) are defined as any untoward medical occurrence that meets one of more of the following criteria:

- Results in death
- Is life-threatening
- Requires inpatient hospitalization or prolongation of existing hospitalization
- Results in persistent or significant disability/ incapacity
