# Supplementary material for: Ventilator-associated pneumonia prevention in the Intensive care unit using Postpyloric tube feeding in China (VIP study): study protocol for a randomized controlled trial
Source: Trials. 2022 Jun 9;23:478. doi: 10.1186/s13063-022-06407-5 (PMC9178536; doi:10.1186/s13063-022-06407-5)
Supplement: Supplementary file 7 — Additional file 7. Informed Consent Form for Participants’ enrolment—English version. [file 13063_2022_6407_MOESM7_ESM.docx]

IRB No:

**Study Title:** Effectiveness of a community health worker delivered care intervention for hypertension control in Uganda: a stepped wedge, cluster randomized control trial.

**Version Date: 24**^th^ September, 2021

| **Investigators** | **Contacts** | **Institution** |
| --- | --- | --- |
| Dr. Robert Kalyesubula  (Principal Investigator) | 0704442700 | Makerere University, Colleges of Health Sciences, Department of Physiology, Kampala, Uganda /ACCESS, Nakaseke, Uganda |
| Juliet Nandawula | 0782276117 | African Community Centre for Social Sustainability (ACCESS), Nakaseke, Uganda. |
| Dr. Richard Munana | 0751702029 | Makerere University, College of Health Sciences, School of Public Health, Kampala Uganda. /ACCESS, Nakaseke, Uganda |
| Ivan Weswa | 0751906332 | ACCESS, Nakaseke, Uganda |

**Background and rationale of the study:**

In Uganda, more than a quarter of the adult population has hypertension. A study carried out in Mukono, Uganda, showed that only 9.4% of adults with hypertension had adequate blood pressure control. Yet, hypertension control is crucial in reducing mortality and morbidity attributable to cardiovascular diseases as well as lowering costs and reducing utilisation of health care resources.

CHWs are an important cadre of the global health workforce as they are involved in providing health services at the community level. CHWs have effectively provided a range of preventive and care interventions for Maternal and Child Health and infections (like HIV, tuberculosis and malaria) in LMICs.

The purpose of the current study is to assess the effectiveness of a community health worker led intervention to control hypertension in Nakaseke district, Uganda. CHWs will, on a monthly basis, visit study participants’ homes to deliver an intervention package that includes healthy lifestyle counselling, screening for risk factors, ensuring medication adherence among others.

You are invited to participate in this research study to enable us achieve the above purpose. Once you understand the study, you will be asked if you wish to participate; if so, you will be asked to sign this form.

**A description of sponsors of the research project and the organisation affiliation of the researchers:**

Doctors from the African Community Centre for Social Sustainability (ACCESS), Makerere University and other universities led by Dr. Robert Kalyesubula are carrying out the study.

**Purpose:**

We are asking for your participation as a volunteer in a research study to help us assess the effectiveness of a CHW-led intervention for the control of hypertension.

**Procedures:**

CHWs will visit you at your home and provide you with a package that includes healthy life style counselling on diet, physical activity, alcohol consumption, medication adherence among others. They will also take physical measurements such as height, weight, blood pressure, and waist circumference. Your blood sugar will also be measured using capillary blood obtained by a finger prick, and analysis of your urine using 10-parameter dipstick will be done. Results of these will be communicate to you immediately.

**Who will participate in the study?**

Hypertensive patients, 18 years and older, attending Nakaseke Hospital and Life Care Center NCD clinics, and residing in either Nakaseke Subcounty, Nakaseke Town Council or Kasangombe Subcounty. 1002 participants will be involved in this study.

**Risks:**

This is a minimal risk study. You will experience some minor discomfort while your blood pressure is being measured or experience some little pain while we measure your blood glucose. There is a possibility that other people not part of the study may know the information you share with us. However, we shall do everything in our powers to secure tightly all your information. If any of your information is used, we will not include your name.

**Benefits:**

This study will help us better understand how effective CHWs can be in the control of high blood pressure.

**Confidentiality:**

All information from this study will be held with strict confidentiality, only the study researchers, local regulatory authorities, and institutional review board/ethics committee may have access to this information.

All the information about yourself and the results of the tests carried out will be collected and stored in a database in password – protected computers. You will not be identifiable, as numbers will be used instead of names in the database. The laboratory staff and the main researcher will know your test results. The Makerere University School of Biomedical Sciences Research and Ethics Committee and the UNCST that have given the researcher permission to conduct this study and the funder/ sponsors that have given the funds to conduct this study may also access your results.

**Cost of participation in the study:**

There is no cost to participate in this research study. You will not be paid for participating in the study. Participation is voluntary. However, you will be compensated with UGX 10,000 for time and effort.

**Compensation for participation in the study:**

Your participation in this study is voluntary. No compensation is available if some injury happens because of your participation in this study.

**Dissemination:**

The results of this study could be presented in journals or scientific conferences, but without your name or any other information that could identify you. Your name will not appear in any publication of this study. Some results might be shared with other investigators; however, this information will not include identification data. The final decision to share this data will be taken by the principal investigators previously mentioned.

**Ethical approval**

The Makerere University School of Biomedical Sciences – Research and Ethics Committee (SBS-REC) an accredited Ugandan based Research and Ethics Committee has given us permission to conduct this study. Please contact or call the chairperson of SBS-REC if you have questions about your rights as a study participant, if you feel you have been treated unfairly or have other concerns. The SBS-REC contact information is:

| Ass.Prof Mwaka Erisa  (Chairperson, SBS-REC) | +256752575050 | Makerere University School of Biomedical Sciences Research and Ethics Committee. |
| --- | --- | --- |

**Assessing understanding**

Feel free to ask anything about the above information for more clarity as well as your rights to participate in the study.

**YOU WILL BE GIVEN A COPY OF THIS FORM THAT YOU MAY KEEP.**

**STATEMENT OF CONSENT FOR PARTICIPATION IN THE STUDY.**

I……………………………………………………………………………………………… acknowledge that the researcher has explained to me the nature, procedure, terms and conditions of this study. I understand why this study is done, what will be done, the risks and benefits as described in this written summary. I understand that my name and address will not appear alongside these samples. I appreciate that my participation is voluntary and that in case I do not participate in, or withdraw from, the study my health will not be compromised.

I here under sign or place my thumbprint as proof of my consent to participate in the study.

Signature of the participant: ………………………………………………………………..

Date: …………………/………………………/…………………………….(DD/MM/YYYY)

**For illiterate volunteers:** I confirm that the information contained in this written consent form has been explained to the participant. He/she has to the best of my knowledge understood the purpose, procedure, risk, and benefits of taking part in the study and has voluntarily agreed to participate in the study.

**For those placing thumbprints only:**

I attest that the participant who states that his /her name is ………………………………………………………………………………………………….has voluntarily placed his/her thumbprint on this consent form on this day …………….………………/……………..…………/ ……………………...(DD/MM/YYYY)

Name of witness to consenting process …………………………………………………………………………………………………..

Signature: ………………………………………………………………………………………

Date: ….../………………/………. ……………………... (DD/MM/YYYY

I have explained the features of this study to the respondent and to the best of my knowledge and conviction he/she has understood its purpose, procedure, benefits and risk that may be involved.

………………………………….. ……………………………. …………………….

Name of study staff Signature Date
